# Periostin Splice Variant Expression in Human Osteoblasts from Osteoporotic Patients and Its Effects on Interleukin-6 and Osteoprotegerin

**DOI:** 10.3390/ijms26030932

**Published:** 2025-01-23

**Authors:** Till Kuebart, Lisa Oezel, Beyza Gürsoy, Uwe Maus, Joachim Windolf, Bernd Bittersohl, Vera Grotheer

**Affiliations:** 1 Department of Orthopedics and Trauma Surgery, Medical Faculty and University Hospital Duesseldorf, Heinrich Heine University, 40225 Duesseldorf, Germany; till.kuebart@uni-duesseldorf.de (T.K.);; 2 Department of Orthopedics, Medical School and University Medical Center Ostwestalen-Lippe (OWL), Klinikum Bielefeld-Mitte, Bielefeld University, 33615 Bielefeld, Germanyvera.grotheer@uni-bielefeld.de (V.G.)

**Keywords:** osteoporosis, periostin, osteoblasts, interleukin-6

## Abstract

Osteoporosis is an inflammatory disease characterised by low bone mass and quality, resulting in weaker bone strength and fragility fractures. Periostin is a matricellular protein expressed in the periosteum of bone by osteoblasts. It regulates cell recruitment and differentiation in response to fracture and contributes to extracellular matrix (ECM) formation. The aim of the following study was to determine the splice variants of Periostin expressed in human osteoblasts and Periostin’s function in the pathophysiology of osteoporosis. Osteoblasts isolated from femoral heads from 29 patients with or without osteoporosis were utilised. Periostin splice variants were compared by quantitative real-time polymerase chain reaction (qPCR). Furthermore, the effect of Periostin inhibition on osteoblast differentiation was investigated using alizarin red S staining. Lastly, the interaction of IL-6 and Periostin and their effect on osteoprotegerin (OPG) secretion were analysed with the implantation of enzyme-linked immunosorbent assays (ELISAs). It could be demonstrated that human osteoblasts preferentially express Periostin isoform 4, even if splice variant expression was not altered in osteoporosis conditions, indicating that Periostin’s functions in bone are primarily attributable to this isoform. The inhibition of Periostin resulted in significantly reduced osteoblast differentiation. However, Periostin was secreted in significantly higher amounts in osteoblasts from patients with osteoporosis. Additionally, Periostin significantly reduces OPG secretion and, thereby, rather promotes bone resorption. Furthermore, it could be determined that Periostin and IL-6 induce each other, and both significantly decrease OPG secretion. A positive feedback loop exacerbates the dysregulation found in human osteoblasts from patients with osteoporosis, thereby contributing to bone loss.

## 1. Introduction

Osteoporosis is an inflammatory disease characterised by low bone mass and inferior bone quality, resulting in weaker bone strength [1,2]. This leads to the occurrence of fragility fractures, predominantly in the hip region, vertebrae, and distal forearm [3].

Osteoporosis is classified by bone mineral density, measured most frequently with dual-energy X-ray absorptiometry (DXA) and stated as the T-Score [4]. The threshold for osteoporosis is set by the World Health Organization (WHO) as 2.5 standard deviations (SDs) below the mean of a young healthy comparison group [5].

In the US in 2010, 10% of adults aged 50 years or older suffered from osteoporosis, and an additional 43.9% exhibited low bone mass [6]. Since osteoporosis is an age-related disease occurring most commonly in postmenopausal women, the prevalence is expected to rise in the coming decades worldwide [7]. Although different therapies are in use, the rate of fractures attributable to osteoporosis remains high [8]. These injuries are not only associated with an increased mortality risk, but also with a growing financial burden on public health, due to the treatment costs and need for long-term care [8].

In osteoporosis, bone resorption by osteoclasts is greater than new bone formation by osteoblasts, resulting in trabecular bone loss. This balance is regulated by the RANK-RANKL-OPG axis [9]. Receptor Activator of NFκB (RANK) is localized on the cell surface of osteoclasts, promoting their activation upon binding of Receptor Activator of NFκB Ligand (RANKL) [10]. Osteoprotegerin (OPG) functions as a decoy receptor, binding to RANKL and trapping it before it binds RANK [10]. Both RANKL and OPG are secreted by osteoblasts [11]. Furthermore, osteoblasts build up bone by matrix deposition and subsequent mineralization, which makes them crucial cells in bone homeostasis [12].

During a lifetime, total bone mass is tightly regulated. Peak bone mass (PBM) is achieved at 18 to 24 years on average, depending on the examined bone and gender [13]. After reaching PBM, it decreases continuously, even without developing manifest osteoporosis, in the female hip by 0.3% every year [14].

Periostin is a matricellular protein expressed in bone [15]. It regulates cell recruitment, migration, and proliferation in the periosteum [16,17]. Additionally, it controls the cell adhesion to extracellular matrix (ECM) components [18] and enhances osteoblast differentiation [19,20]. The self-renewal of the periosteal stem cells’ niche is Periostin-dependent [17].

Periostin regulates collagen cross-linking and the formation of an extracellular meshwork [21]. Periostin binds to fibronectin via its elastin microfibril interface (EMI) domain, anchors itself to the Golgi apparatus, and facilitates the binding of fibronectin to tenascin-C [22,23]. This promotes the secretion of both proteins, with the subsequent detachment of Periostin, enabling the formation of fibrils, which are a prerequisite for collagen fibres [24]. Furthermore, Periostin promotes the proteolytic activation of lysyl oxidase (LOX) [25], which in turn catalyses the cross-links of collagen fibres [26].

Periostin has been known to be induced by transforming growth factor β (TGFβ) [15]. Its expression is also enhanced by the Th2-Cytokines Interleukin-4 (IL-4) and Interleukin-13 (IL-13) in lung fibroblasts, which trigger inflammatory responses [27]. IL-6 also induces Periostin in fibroblasts in colorectal tumorigenesis [28]. Taken together, these findings indicate a function of Periostin in initiating inflammation, which has not yet been fully understood.

Bone healing can be divided into different phases, known as the inflammatory, repair, and remodelling phases [29]. The inflammatory phase is controlled by immune cells that secrete pro-inflammatory cytokines, including IL-6, that activate the periosteum [30,31]. It is an essential part of fracture healing; however, chronic inflammation leads to impaired and prolonged fracture healing [30,32]. In the inflammatory phase, Periostin is secreted within the periosteum, and during subsequent callus formation, it is secreted by osteoblasts on new bone trabeculae [17]. Accordingly, during the process of bone remodelling, Periostin expression occurs in different locations and at different times.

Osteoblasts in bone tissue secreting Periostin can thus regulate bone formation in the event of injury or increased stress. Across divergent tissues, Periostin has a similar role. The baseline expression is relatively low and is upregulated after injury [33,34]. In chronic diseases like fibrosis, pathologically high levels of Periostin could be detected, and therapeutic improvement could be reached upon inhibition of Periostin [33].

The Periostin C-terminal region acts as a heparin-binding site [35], and it can be alternatively spliced, forming different isoforms [15]. Ten different isoforms are known today (see Table S1) [36]. Periostin isoforms are tissue-specific and can serve different functions, for example by enhanced integrin binding [37,38], but their impact on osteoporosis is not yet known. Alternative splicing changes the characteristics of the protein so that it modulates the function of other domains [39,40]. Deletion of the entire C-terminal region leads to enhanced binding of Periostin to fibronectin, tenascin-C, and collagen type V, suggesting that it regulates the binding of the Fasciclin-1 (FAS1) domains [27], which are responsible for the binding to bone morphogenetic protein 1 (BMP-1), tenascin-C, and integrins αvβ3 and αvβ5 [23,41]. Periostin splice variant expression can be regulated by TGFβ [15] as well as IL-4 and IL-13 [27]. In lung fibroblasts, after stimulation with IL-4 and IL-13, three different protein sizes were observed; the dominating isoform was identified as Periostin-4 [27].

Osteoimmunology describes the effect the immune system has on bone metabolism [42]. It is known today that these two systems are closely linked together and influence each other [2]. Moreover, aging, a major risk factor for osteoporosis, is associated with a low-grade, chronic, systemic inflammatory state, termed inflammaging [43]. In osteoporosis, the IL-6 expression is elevated in bone [44]. Also, other chronic inflammatory diseases lead to increased bone resorption, such as rheumatoid arthritis and Crohn’s disease [45,46].

The aim of this study was to further elucidate the mechanisms behind the pathophysiology of osteoporosis, focusing on the effect of Periostin on osteogenic differentiation and inflammatory bone reactions. For that, which kind of Periostin splice variant is preferably expressed in human osteoblasts should be analysed. Another hypothesis for investigation was to determine whether a discrepancy exists between osteoblasts from patients with and without osteoporosis.

## 2. Results

### 2.1. Expression Pattern of Periostin Messenger Ribonucleic Acid (mRNA) Splice Variants in Osteoblasts

To determine the mRNA expression pattern of Periostin and its splice variants and the effects of cytokines on the expression, normalisation to a housekeeping gene control was required.

**Figure 1:**
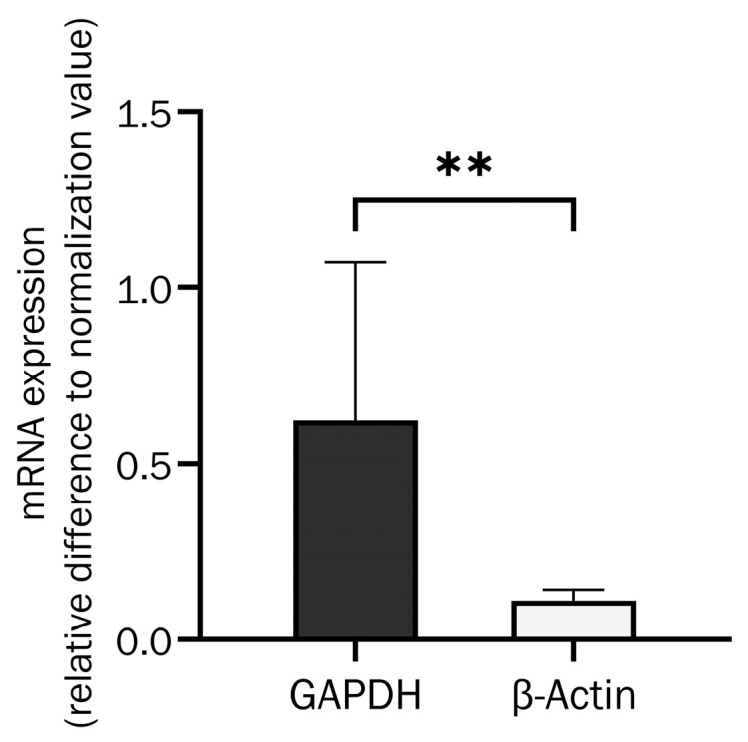
Comparison of housekeeping gene mRNA expression variance when osteoblasts stimulated with IL-6 (250 U/mL), IL-8 (100 U/mL and 25 U/mL), and their inhibitors (MA5-23698 at 0.1 µg/mL and Reparixin L-lysine salt at 100 nM) (unpaired *t*-test with Welch’s correction, t = 3.419, df = 8.083, *p* = 0.009 (**), n = 10).

To evaluate the optimal housekeeping gene for human primary osteoblasts, the expression of glyceraldehyde 3-phosphate dehydrogenase (GAPDH) and β-actin was compared in two osteoblast donors and with stimulation with IL-6, IL-8, and their inhibitors at different concentrations. The results are shown in Figure 1 as the relative change from the normal value without cytokines. There was a significantly lower variance of β-actin expression, at approximately 19%, compared to GAPDH expression.

β-Actin is, therefore, superior to GAPDH as a housekeeping gene control for primary human osteoblasts and cytokine stimulation. Consequently, qPCR results have been normalised to the corresponding β-actin control.

**Figure 2:**
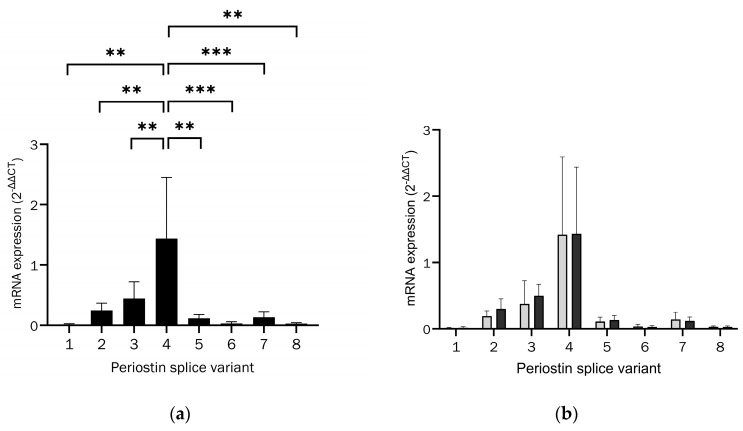
(**a**) mRNA expression of Periostin splice variants in osteoblasts relative to total Periostin expression (outliers of ROUT with Q = 1% excluded, Kruskal–Wallis test, Kruskal–Wallis statistic = 97.12, *p* < 0.01 (**), *p* < 0.001 (***), n = 16, with post hoc analysis by Wilcoxon matched-pairs signed rank test with manual correction of the α-error according to Bonferroni, *p* < 0.0035). (**b**) Comparison of mRNA expression of Periostin splice variants in osteoblasts relative to total Periostin expression between osteoporosis, displayed in black bars, and normal bone density, displayed in light grey bars (outliers of ROUT with Q = 1% excluded, multiple unpaired *t*-tests with Welch’s correction, each *p* > 0.15, n = 15).

Periostin splice variant expression in osteoblasts was conducted using primers established by Cai et al. [47]. The expression pattern is shown in Figure 2a. The full set of data points can be seen in Figure S1a.

The expression differed significantly between the individual Periostin splice variants. The post hoc analysis revealed a significantly increased expression of the splice variant Periostin-4 with the accession number NM_001135936.1 in comparison to all other splice variants. The frequency of expression was found to be more than three times that of isoform 3, and more than 10-fold that of variants 1 and 5–8.

The expression of splice variants in patients with osteoporosis was found to be like that observed in patients with normal bone density, with Periostin isoform 4 identified as the dominant splice variant. The mRNA expression of the individual splice variants did not exhibit a significant difference depending on the T-value, as illustrated in Figure 2b. The full set of data points can be found in Figure S1b.

### 2.2. Periostin Expression and Secretion in Osteoporosis

To further elucidate the function of Periostin in osteoporosis, the expression levels of Periostin were examined in relation to bone density.

**Figure 3:**
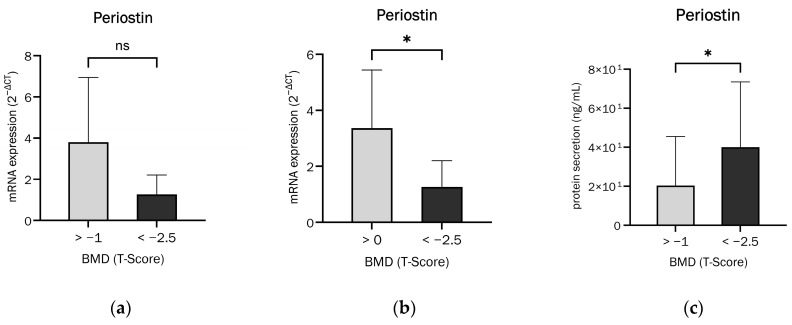
(**a**) Comparison of Periostin mRNA expression (relative to housekeeping gene) between normal bone density and osteoporosis patients (unpaired *t*-test with Welch’s correction, t = 2.034, df = 7.065, *p* = 0.081 not significant (ns), n = 14). (**b**) Comparison of Periostin mRNA expression (relative to housekeeping gene) between patients with T-Score > 0 and <−2.5 (unpaired *t*-test, t = 2.378, df = 10, *p* = 0.0388 (*), n = 12). (**c**) Comparison of Periostin secretion between normal bone density and osteoporosis patients (Mann–Whitney test, U = 27, *p* = 0.0281 (*), n = 22).

Figure 3a presents a comparison of Periostin mRNA expression between patients with regular bone density and osteoporosis, as defined by clinical bone density limits. The Periostin mRNA expression in osteoblasts from osteoporosis patients was observed to be, non-significantly, two-thirds lower than that of patients with normal bone density.

However, if T-values greater than 0 and less than −2.5 were established, the expression of Periostin mRNA was found to be significantly lower in osteoblasts derived from patients with osteoporosis compared to those with a bone density T > 0, as shown in Figure 3b.

The isolated expression of the most frequently formed splice variant, Periostin isoform 4, also follows this trend on the mRNA level, although the result is not statistically significant (see Figure S2).

Periostin mRNA expression and bone density can be investigated through a linear regression analysis; see Figure S3. The linear regression model revealed a non-significant but strong correlation along a straight line according to the effect size measure R^2^. Therefore, it can be estimated that the lower the bone surface density, the less Periostin mRNA is expressed.

The protein expression of Periostin was analysed to control for the trend observed at the mRNA level. The relative Periostin protein expression was found to be statistically not significantly different between compared groups, as illustrated in Figure S4. Nevertheless, the observed trend of elevated Periostin protein expression in osteoblasts of patients with regular bone density, in comparison to those with osteoporosis, is also apparent in this context.

Given the established characteristics of Periostin in relation to the ECM, an enzyme-linked immunosorbent assay (ELISA) analysis was conducted to assess the secretion of Periostin into the ECM [22,23].

We found that Periostin secretion was significantly increased in osteoblasts from patients with osteoporosis, as shown in Figure 3c. The previously demonstrated trend for Periostin mRNA and protein expression was reversed for secretion, with osteoblasts from osteoporotic patients secreting 97% more Periostin.

### 2.3. Periostin Expression and Secretion in Relation to Age

To identify a corresponding change in Periostin in relation to bone mass loss with advancing age [13], mRNA expression and secretion were also analysed according to patient age.

**Figure 4:**
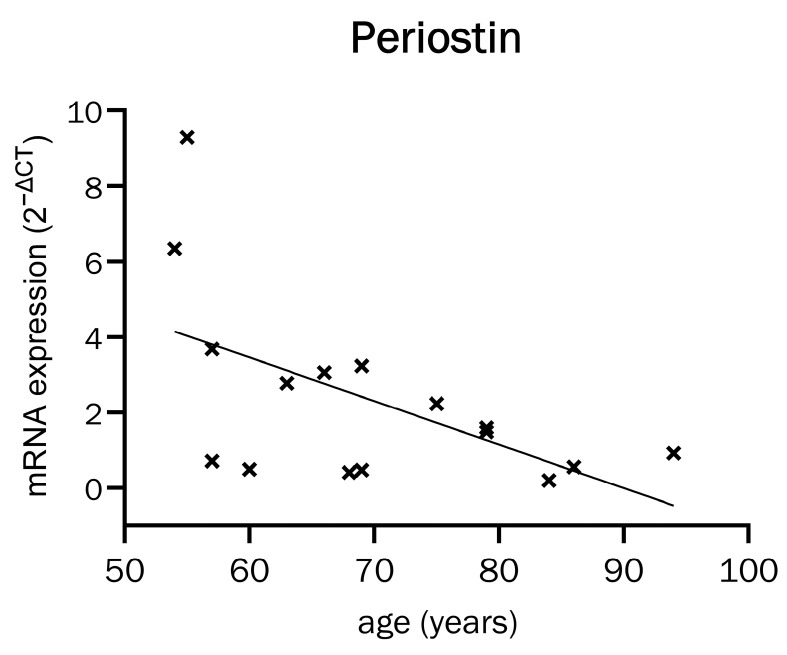
Periostin mRNA expression in relation to patient age (simple linear regression, y = −0.04480x + 4.029, R^2^ = 0.3199, *p* = 0.0224, n = 16).

There is a strong correlation between Periostin mRNA expression in osteoblasts and patient age. Figure 4 shows a notable decline observed with increasing age.

Furthermore, Periostin secretion exhibits a non-significant decline with advancing age, with a comparatively diminished effect size (see Figure S5, simple linear regression, y = −1.475x + 157.8, R^2^ = 0.2349, *p* = 0.1309, n = 11). The collective findings illustrate a correlation between Periostin and patient age, which may contribute to the expression of Periostin in osteoporosis.

### 2.4. Effect of IL-6 on Periostin Secretion

The precise role of Periostin in bone during inflammation remains unclear. To investigate this question, the alteration in Periostin secretion following stimulation with the proinflammatory cytokine IL-6 and an IL-6 antibody (MA5-23698) was examined [48]. Protein secretion was evaluated through ELISA after a 21-day differentiation period.

**Figure 5:**
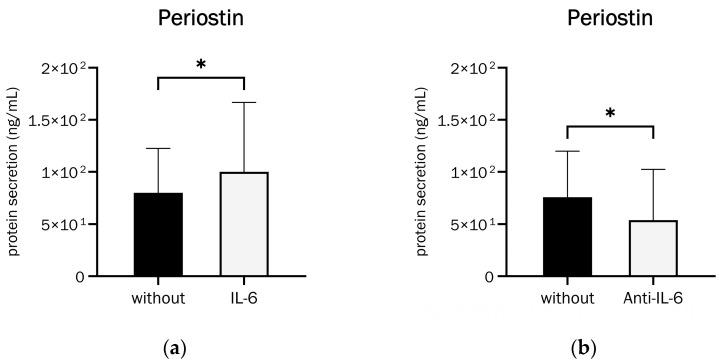
Effect of (**a**) IL-6 (250 U/mL) and (**b**) anti-IL-6 (MA5-23698 at 0.1 µg/mL) on Periostin secretion in 21-day-differentiated osteoblasts ((**a**): Wilcoxon matched-pairs signed rank test, *p* = 0.0425 (*), n = 12, (**b**): Wilcoxon matched-pairs signed rank test, *p* = 0.0134 (*), n = 14).

Figure 5 shows that IL-6 significantly increased Periostin secretion, by approximately 25%. The addition of anti-IL-6 had the opposite effect and inhibited Periostin secretion by approximately 29%. IL-6 is, thus, a capable stimulating factor for Periostin secretion in human osteoblasts.

Periostin mRNA expression showed a non-significant increase of approximately 21% upon stimulation with IL-6, as shown in Figure S6.

### 2.5. Interacting Effect Between Periostin and IL-6

**Figure 6:**
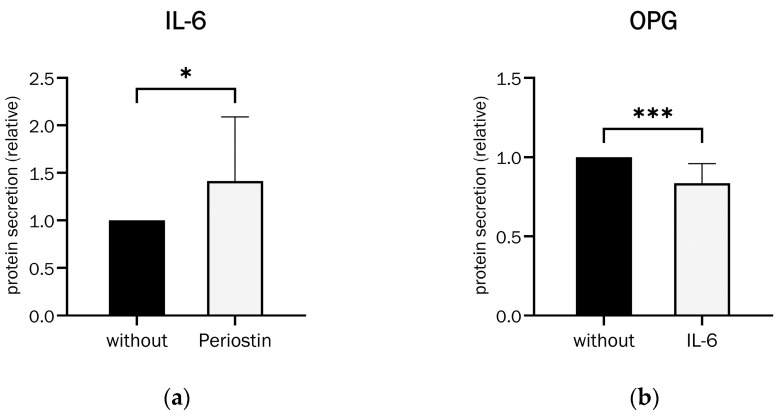
(**a**) Effect of Periostin (100 ng/mL) on IL-6 secretion in 21-day-differentiated osteoblasts (Wilcoxon matched-pairs signed rank test, *p* = 0.0479 (*), n = 13). (**b**) Effect of IL-6 (250 U/mL) on OPG secretion in 21-day-differentiated osteoblasts (Wilcoxon matched-pairs signed rank test, *p* = 0.0004 (***), n = 14).

Additionally, the effect of Periostin on IL-6 secretion was determined. Figure 6a shows the approximately 42% significant increase in IL-6 secretion by osteoblasts after Periostin stimulation.

In turn, IL-6′s effect on OPG secretion was investigated. IL-6 was found to significantly inhibit OPG secretion by a median of 14% (see Figure 6b).

These findings illustrate the effects of a possible positive feedback loop between Periostin and IL-6 on osteoporosis; Periostin’s effect on OPG secretion is shown in Section 2.7.

### 2.6. Effect of Cytokines on Periostin Splice Variant mRNA Expression

In addition to the effect of cytokines such as IL-6 on the formation of total Periostin, the impact on the splice variants formed is of interest in assessing the function of Periostin in bone during inflammation and osteoporosis.

**Figure 7:**
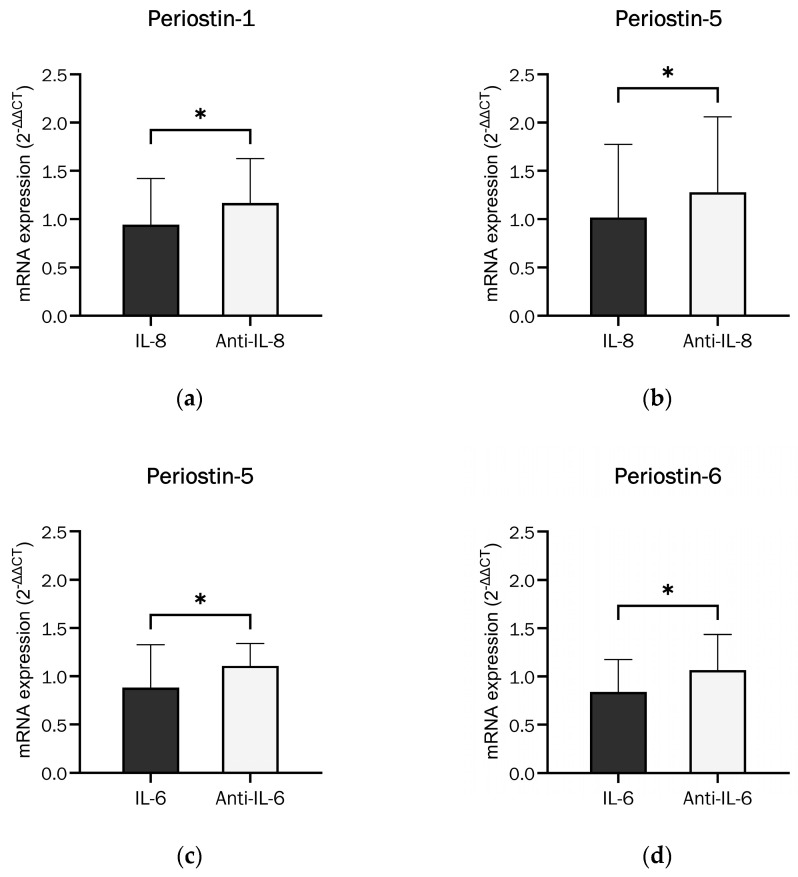
Effects of cytokines and their inhibitors on the Periostin splice variant mRNA expression in undifferentiated osteoblasts. (**a**) Effect of IL-8 (100 U/mL) and its inhibitor (Reparixin L-lysine salt at 100 nM) on Periostin isoform 1 mRNA expression (paired *t*-test, t = 2.324, df = 13, *p* = 0.037 (*), n = 14). (**b**) Effect of IL-8 (100 U/mL) and its inhibitor (Reparixin L-lysine salt at 100 nM) on Periostin isoform 5 mRNA expression (Wilcoxon matched-pairs signed rank test, *p* = 0.0107 (*), n = 14). (**c**) Effect of IL-6 (250 U/mL) and anti-IL-6 (MA5-23698 at 0.1 µg/mL) on Periostin isoform 5 mRNA expression (paired *t*-test, t = 2.442, df = 13, *p* = 0.0297 (*), n = 14). (**d**) Effect of IL-6 (250 U/mL) and anti-IL-6 (MA5-23698 at 0.1 µg/mL) on Periostin isoform 6 mRNA expression (Wilcoxon matched-pairs signed rank test, *p* = 0.0245 (*), n = 14).

Individual splice variants were differentially expressed if IL-6 or IL-8 and the corresponding inhibitor were added, as shown in Figure 7. Periostin-1 and -5 were significantly less expressed with the addition of IL-8 compared to the inhibition of IL-8 (Reparixin L-lysine salt). IL-6 and anti-IL-6 (MA5-23698) had a similar effect on the Periostin-5 and -6 splice variants. Periostin-5 was thus the only splice variant that responded significantly to both IL-6 and IL-8.

### 2.7. Effect of Periostin on Relevant Osteoporosis Parameters

**Figure 8:**
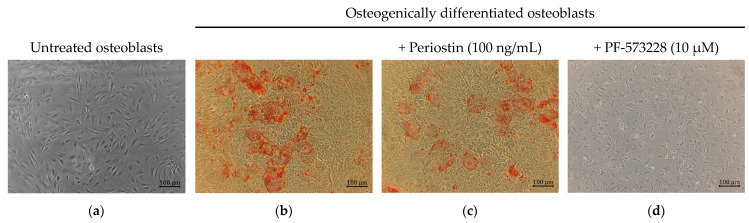
(**a**) Representative osteoblasts after two days of incubation without differentiation, (**b**) after 21 days of differentiation without additives, (**c**) after 21 days of differentiation with Periostin (100 ng/mL), and (**d**) after 21 days of differentiation with Periostin inhibition (PF-573228 at 10 µM), all visualized with alizarin red S staining.

The effect of Periostin on osteogenic differentiation was investigated by alizarin red S staining of the calcium deposition of osteoblasts. Controls without other additives were compared with Periostin inhibition. Figure 8 shows representative images under these conditions.

**Figure 9:**
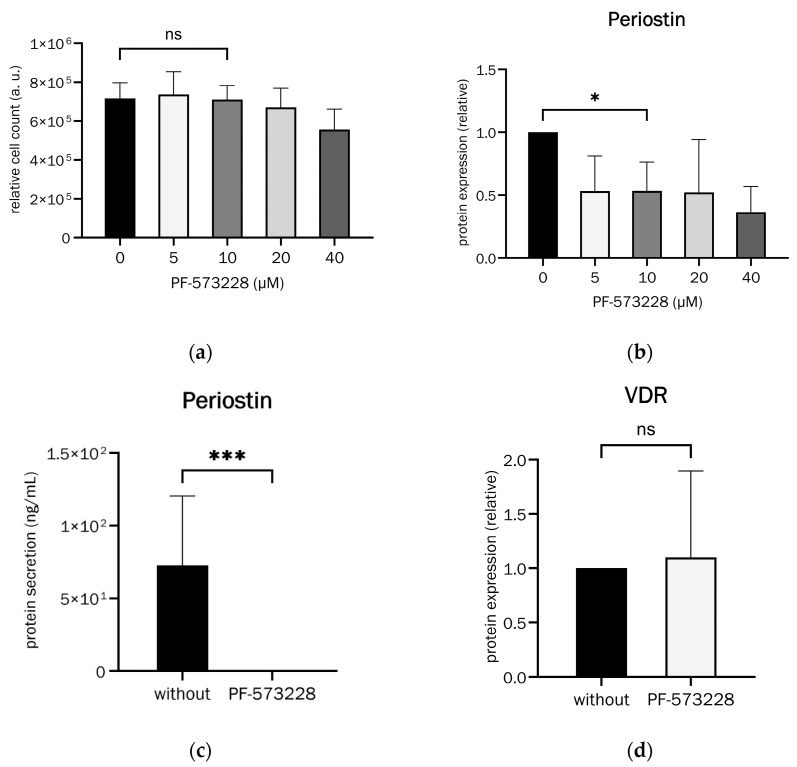
(**a**) Effects of PF-573228 concentrations on undifferentiated osteoblast viability (paired *t*-test, t = 0.2366, df = 2, *p* = 0.835 (ns), n = 3). (**b**) Effect of PF-573228 concentrations on Periostin protein expression in undifferentiated osteoblasts (paired *t*-test, t = 4.549, df = 4, *p* = 0.0104 (*), n = 5). (**c**) Effect of PF-573228 on Periostin secretion in 21-day-differentiated osteoblasts (Wilcoxon matched-pairs signed rank test, *p* = 0.001 (***), n = 11). (**d**) Effect of PF-573228 on Vitamin D receptor (VDR) expression in 21-day-differentiated osteoblasts (Wilcoxon matched-pairs signed rank test, *p* = 0.7334 (ns), n = 12).

In Figure 9, the FAK inhibitor PF-573228 effectively and significantly suppressed Periostin secretion without reducing cell viability at 10 µM.

Furthermore, osteoblasts had not yet differentiated into osteocytes when stimulated with PF-573228 (see Figure 9d). For that, Vitamin D receptor (VDR) expression was analysed as a marker for osteoblasts since it is not expressed anymore in osteocytes [49], and we found no significant change in its expression.

**Figure 10:**
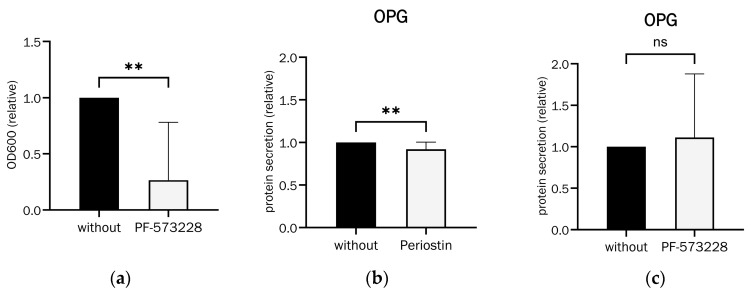
Effects of (**a**) Periostin inhibition (PF-573228 at 10 µM) on osteoblast differentiation, analysed with alizarin red S staining (Wilcoxon matched-pairs signed rank test, *p* = 0.0039 (**), n = 10) and (**b**) Periostin (100 ng/mL) and (**c**) Periostin inhibition (PF-573228 at 10 µM) on OPG secretion in 21-day-differentiated osteoblasts ((**b**): Wilcoxon matched-pairs signed rank test, *p* = 0.004 (**), n = 14, (**c**): Wilcoxon matched-pairs signed rank test, *p* = 0.5693 (ns), n = 12).

Osteogenic differentiation with Periostin inhibition by the FAK inhibitor PF-573228 was significantly reduced compared to cells without Periostin inhibition, as seen in Figure 10a.

Effective inhibition of Periostin secretion, therefore, results in a significant reduction in matrix mineralization as a measure of successful osteogenic differentiation.

OPG secretion with the addition and inhibition of Periostin was used to demonstrate its effect on relevant osteoporosis parameters. OPG secretion was significantly reduced by approximately 8% upon the addition of Periostin. The addition of the FAK inhibitor PF-573228 increased it non-significantly by an average of approximately 11%, both shown in Figure 10b,c.

## 3. Discussion

Periostin is a matricellular protein expressed in the periosteum of bone [15]. Although its role in bone-regulating cells and the formation of an extracellular meshwork is relatively known [16,17,21], there is little evidence for the effects of Periostin in the pathogenesis of osteoporosis. Since Periostin is secreted by osteoblasts, and these have a substantial impact on bone homeostasis and osteoporosis, we investigated the expression of Periostin in human osteoblasts and its effects on osteogenic differentiation as well as OPG and IL-6 secretion.

### 3.1. Periostin Splice Variants in Human Osteoblasts

This study was the first to analyse the expression of Periostin splice variants in primary human osteoblasts, especially from aged donors. As shown in Figure 1, we used β-actin for normalisation, because GAPDH fluctuates in osteoblasts, especially if incubated with cytokines. All examined splice variants were detected, and the Periostin-4 splice variant was dominantly expressed, even if splice variant Periostin-3 was also expressed at relevant levels. This differs from splice variant expression in chondrocytes and breast cancer [47,50], suggesting a cell type-specific expression and function of Periostin. In periodontal ligament cells, Periostin-4 was also upregulated [37].

Exons 17 and 21 are missing in both Periostin-3 and -4 [36], suggesting that this feature is relevant to the function of Periostin in osteoblasts. The absence of exon 21 has been shown to increase binding to tenascin-C, which is secreted in a complex with Periostin and fibronectin into the ECM [23].

No change in the expression pattern of Periostin splice variants was detected in osteoblasts from osteoporosis patients compared to osteoblasts from patients with normal bone density. This indicates that alternative splicing of Periostin has no special function in the pathogenesis of osteoporosis. These results are contrary to other studies, where, for example, in retinal neovascularisation, Periostin exons 17 and 21 have been identified as contributing to the pathogenesis [51], and in bladder cell carcinoma, several Periostin splice variants are upregulated [38].

Increased expression of IL-6 in patients with osteoporosis was detected [44]; therefore, the effect of cytokines on Periostin splice variant expression was evaluated here. For individual splice variants, modulation of expression by IL-6 and/or IL-8 was demonstrated. Cytokines, therefore, have an effect, albeit small, on the expression pattern of splice variants in osteoblasts. However, the dominantly expressed variant 4 was not one of them, so the impact of these expression changes cannot be classified here. Additionally, the effect of cytokines on the expression of splice variants did not differ significantly in osteoblasts from patients with osteoporosis.

### 3.2. Periostin Expression in Old Age

A significant correlation was found between donor age and Periostin expression. The older the patient, the less Periostin mRNA was expressed by the osteoblasts.

Our patient cohort was aged between 54 and 94 years. A significant decrease in serum Periostin has already been shown to occur from 16–18 years to 30–32 years [52], i.e., from the time towards the end of bone growth to the time when peak bone mass is reached. We were able to demonstrate this correlation at the cellular level and extend it to older age, when bone density typically degrades [13].

Age is the greatest risk factor for osteoporosis [53]. Therefore, the significant correlation of Periostin with age suggests at least an association with osteoporosis. However, it is not possible to conclude from our finding alone whether Periostin mRNA decreases with age as a cause or consequence of osteoporosis. Age might be a possible confounding factor causing both low bone mineral density and low Periostin expression.

### 3.3. Periostin Expression and Secretion in Osteoporosis

The expression of both Periostin mRNA and protein was significantly, or at least in tendency, increased in osteoblasts from patients with normal bone density compared to osteoblasts from patients with osteoporosis. Excitingly, this relationship was reversed when monitoring Periostin secretion, since osteoblasts from osteoporosis patients secreted significantly more Periostin.

This observation is entirely new. A post-translational modification of Periostin that affects its secretion is conceivable. The vitamin K-dependent γ-carboxylation of Periostin would be one possibility [54]. It is also known that the plasma concentration of vitamin K and bone density correlate significantly in men and postmenopausal women [55]. Lower vitamin K availability, depending on bone density, could, therefore, lead to reduced γ-carboxylation, resulting in enhanced secretion. For factor IX, a vitamin K-dependent coagulation factor, it has been shown that secretion is significantly reduced when vitamin K-dependent γ-carboxylase is overexpressed [56]. A similar mechanism may occur in osteoblasts in bone, but this has not yet been investigated.

A cleavage of certain regions is also possible. It has been shown in fibroblasts that the heparin-binding site at the C-terminus of Periostin can be proteolytically cleaved after myocardial infarction [40]. The extent to which these modifications may affect secretion is not clarified in detail. However, proteolytic cleavage of the C-terminus allows increased binding to tenascin-C and, thus, improved collagen cross-linking [23].

The reason for the altered expression-to-secretion ratio between osteoblasts from patients with regular bone density and osteoporotic individuals remains unclear. As Periostin exerts its main effect extracellularly via collagen cross-linking [21,23], binding to integrin receptors [57], and as a matricellular protein [58], we will focus on Periostin secretion as the key parameter.

### 3.4. Dependency of Osteoblast Differentiation on Periostin

The increased secretion of Periostin in osteoblasts from donors with osteoporosis suggests a role for Periostin in the pathogenesis of the disease. We have, therefore, evaluated the role of Periostin in osteoporosis in the cell culture model.

Osteogenic differentiation and ECM formation could not or only partially take place when Periostin was inhibited. This has been previously demonstrated for osteoblasts obtained from neonatal rats and the mouse osteoblast cell line MC3T3-E1 [19,20]. We were able to confirm this for primary human osteoblasts. Periostin thus plays an essential role in the differentiation of human osteoblasts.

### 3.5. Effect of Periostin on Inflamed Bone

We were able to demonstrate that Periostin reduced OPG secretion in osteoblasts in our study. This would reduce the OPG/RANKL ratio and thus shift bone homeostasis towards an increased degradation. We hypothesise that this may be part of the bone’s physiological response to fracture and Periostin’s role in bone regeneration.

Periostin has different roles in bone regeneration depending on the stage and location. The initial inflammatory phase is regulated by cytokines [59]. In this phase, Periostin is secreted in the activated periosteum [17]. Here, Periostin could have a regulatory role and initially promote the removal of bone damage by inhibiting OPG secretion. This is supported by our finding that Periostin secretion is stimulated by IL-6, which is important in this phase of bone regeneration [31]. TGFβ also stimulates Periostin and is a well-known wound-healing parameter [60]. In addition, it is known from experiments with Periostin knockout mice that although they do not exhibit a pathological phenotype at birth [61], bone healing is impaired; they display decreased callus formation and bone volumes after fracture [17]. In the absence of Periostin, bone defects are not regenerated but persist for more than 30 days, and bone strength is limited in the long term [23,62]. This suggests that Periostin controls the resorption of the defect via OPG secretion during the inflammatory phase of fracture.

During subsequent callus formation, Periostin is secreted by osteoblasts at new bone trabeculae [17]. Here, Periostin may exert its bone-building function as a promoter of the ECM network and control osteoblast differentiation, as we have demonstrated.

In the pathogenesis of osteoporosis, these mechanisms of bone regeneration could be dysregulated, resulting in pathological, permanently increased secretion of Periostin. As shown, this would then lead to reduced OPG secretion and shift the OPG/RANKL ratio towards bone resorption. If this occurs in the long term, bone density decreases, and osteoporosis development could be supported.

We propose that the dysregulation of Periostin secretion in osteoporosis is caused by chronic inflammation in osteoporosis [2,44,63]. TGFβ is known to promote Periostin expression in osteoblasts [15]. IL-6 also stimulates Periostin secretion in osteoblasts, as demonstrated in our study. Periostin in bone is, thus, closely linked to inflammation, suggesting sequential expression during fracture healing and pathologically increased expression during chronic inflammation in osteoporosis.

We were also able to show that Periostin secretion is not only stimulated by IL-6, but also promotes IL-6 secretion itself. This suggests an autocrine positive feedback loop in bone between Periostin and osteoblast-derived IL-6. This feedback loop has already been demonstrated for chondrocytes in animal models and is mediated by signal transducer and activator of transcription 3 (STAT3) and integrin αvβ3 [64]. Similarly, recent studies have shown that Periostin and TGFβ form a positive feedback loop in fibroblasts and in hepatocellular carcinoma [65,66].

These findings suggest that chronic inflammation is associated with pathologically increased Periostin secretion in bone. This overexpression has tissue-specific effects. In skin wound healing, Periostin expression is also spatio-temporally regulated and limited to the duration of wound healing [67]. However, overexpression results in prolonged wound healing [67] and hypertrophic scar formation [68]. In bone, it may play a crucial role in the pathogenesis of osteoporosis.

To gain a better understanding of the interplay between Periostin and IL-6 in the pathogenesis of osteoporosis, the effect of IL-6 in human osteoblasts was investigated. We were able to confirm the bone-resorbing effect IL-6 exhibits in mice cell cultures [69,70] for human osteoblasts. IL-6 is known to trigger bone resorption via increased RANKL secretion [71]. We have shown that IL-6 also affects OPG secretion in osteoblasts and significantly reduces it. This explains the effect of IL-6 on the OPG/RANKL ratio by a further mechanism and confirms the bone-reducing effect. Furthermore, in response to mechanical stress, both IL-6 and Periostin are upregulated in osteoblasts [72,73]. In ligamentum flavum, increased IL-6 expression in response to mechanical stress is mediated by Periostin [74]. More research is needed to clarify this dependency, most likely by determining OPG secretion during combined stimulation with IL-6 and a Periostin inhibitor or vice versa.

### 3.6. The Role of Periostin in the Pathogenesis of Osteoporosis

Based on our findings, Periostin may play a crucial role in the pathogenesis of osteoporosis. Although Periostin expression is reduced, its secretion is increased, likely due to chronic inflammation of the bone in osteoporosis. As presented in our results, Periostin itself increases inflammation through cross-induction with IL-6. At the same time, Periostin inhibits OPG secretion and may, therefore, contribute to bone resorption in osteoporosis.

This bone-resorbing function of Periostin is in contrast to previous findings that demonstrated no effective bone repair in Periostin knockout mice [62]. In addition, we have shown that Periostin is essential for osteoblast differentiation. The increased secretion with reduced expression may also indicate that the dysregulation of secretion is independent of the development of an effective ECM. This means that secretion may occur without binding to tenascin-C and fibronectin, so that the secreted Periostin does not contribute to the formation of the ECM.

Kudo (2017) postulates a similar phenomenon of alternating functions of Periostin in osteoblasts of medaka fish [75]. Here, two isoforms of Periostin of different lengths are expressed [76], with osteoblast differentiation being inhibited by the short isoform and stimulated by the long isoform [75]. Kudo refers to this as a ‘Periostin switch’, in which Periostin initially assumes regulatory functions and later promotes collagen cross-linking by proteolytic cleavage of the C-terminus [23,75]. Such a switch may be causally involved in the pathogenesis of osteoporosis. Although in our study, no different isoforms of Periostin were detected in osteoblasts from osteoporotic donors, another method of post-translational modification could lead to a modified function of this protein. The implications of this are that Periostin is essential for osteoblast differentiation and ECM buildup, but at a different time in bone regeneration, and in osteoporosis, it acts as a regulator of bone mass by suppressing OPG secretion.

### 3.7. Clinical Applications

Periostin could be of diagnostic and therapeutic importance.

Periostin has been identified as an effective serum biomarker for several diseases, most recently for pulmonary diseases such as asthma [77,78]. According to current knowledge and our understanding of the function of Periostin, it should be detectable at elevated levels in acute fracture healing and osteoporosis compared to an age-adjusted normal value. An age-dependent interpretation of serum Periostin levels is probably also necessary because of the age-dependent expression of Periostin that we have demonstrated. Although no correlation between serum Periostin and bone density has been demonstrated in previous studies [52], an elevated serum Periostin level has been associated with an increased risk of low-trauma radius fractures [79], suggesting a relationship with osteoporosis in line with our observations. Because of its interaction with IL-6, Periostin could also potentially be used as a marker of chronic inflammation in patients with known osteoporosis, to allow a causal therapeutic approach.

Interrupting the vicious cycle between Periostin and IL-6 or TGFβ could be a potential target for new therapeutics in osteoporosis. It has already been shown that the addition of tocilizumab, a monoclonal antibody against IL-6, to the treatment of rheumatoid arthritis significantly increases bone density in patients with osteoporosis [80]. Tocilizumab has also been shown to reduce inflammation and bone resorption in periodontitis [81]. Not only its effect on bone density in osteoporosis, but also on Periostin serum levels, would be of interest.

Possibly, inhibition of Periostin could achieve the same therapeutic benefit. There are no approved drugs for the direct inhibition of Periostin. However, anti-Periostin antibodies have been established in mouse and cell culture models and have shown promising results in other diseases. Anti-Periostin antibodies were able to significantly reduce fibrosis in chronic kidney disease in the cell culture of a mouse model by altering inflammatory and TGFβ signalling pathways [82]. In bleomycin-induced lung fibrosis in mice, Periostin antibodies were able to prolong survival [83]. An anti-Periostin antibody for osteoporosis could exploit the expression of splice variants in bone and target Periostin-4 and -3. This would allow much more specific inhibition without blocking the function of Periostin in other tissues. An anti-exon 17 antibody has been successfully used in rats to preserve cardiac function after myocardial infarction [84]. Nevertheless, further research is needed to clarify whether a Periostin inhibition would yield a net gain of bone mass since a total blockade would prevent osteoblasts from differentiating and bone regeneration from taking place.

The effect of Periostin could also be inhibited via its receptor or downstream in the signalling cascade. However, clinical trials with cilengitide, an integrin αvβ3 and αvβ5 inhibitor, or abituzumab, an anti-integrin αv antibody, have not been promising in other diseases [85].

Valsartan, an established drug, is used as an antihypertensive, and has been shown to suppress Periostin in the heart muscle [86,87]. If a similar effect occurs in bone, valsartan could be used for therapeutic purposes more quickly than previously untrialled drugs.

In addition to anti-inflammatory effects, the aim may be to promote physiological inflammatory regulation. This is achieved by so-called specialised pro-resolving mediators (SPMs) [88]. In bone, resolvin E1 (RvE1) has been identified as bone-sparing [89]. RvE1 acts by reducing osteoclast differentiation and IL-17-mediated RANKL expression by osteoblasts [90]. There is as yet no SPM with an effect on IL-6 and Periostin-mediated bone resorption. This would be dysregulated in osteoporosis with increased IL-6 and Periostin expression and could also be used therapeutically to slow inflammation and bone loss.

### 3.8. Limitations of the Study

We categorised the patient group according to bone density to draw conclusions about osteoporosis status. It should be noted that the patients with normal bone density were not healthy and received a hip replacement for other reasons. These were mostly osteoarthritis, or traumata of the femoral head. Their effects on our findings regarding inflammation and the interplay between Periostin and IL-6 are unclear.

Subjects were categorised according to DXA measurement. This is a method of measuring bone surface density that is inferior to volumetric bone density measurement using qCT for the diagnosis of manifest osteoporosis [91]. However, as it is the clinical gold standard for the diagnosis of osteoporosis [92], it allows the strongest conclusions to be drawn about this patient group.

Our results are based on primary cell cultures of human osteoblasts from elderly patients. This has the advantage that we do not have to make any assumptions about the extrapolation of our findings from other organisms to humans, and from younger patients to a disease most commonly occurring in the elderly. However, we have consistently found relatively large differences between individual donors, suggesting factors that vary the physiology of bone, but are unknown to date.

In addition, the full physiology of bone and osteoporosis as a systemic chronic disease cannot be reproduced in an in vitro cell culture of cancellous bone osteoblasts. This is true in the case of this study, where we observed an alignment of the results comparing osteoblasts from patients with normal bone density and osteoporosis the longer the cell culture differentiation took place. The impact of our experiments on osteoclasts and the resulting interaction with osteoblasts remains uncertain. In addition, mechanical stress, which is important for osteoblasts [72] and Periostin expression [74], was absent in our cell culture.

Lastly, to fully elucidate the role of Periostin in the physiology of bone, its interplay with RANKL could be of interest. However, mature osteoblasts secrete only minimal quantities of RANKL, rendering a meaningful statistical analysis unfeasible. This is because mature osteoblasts predominantly express OPG, whereas early osteoblasts are primarily responsible for RANKL expression [93]. For this reason, we focused on the effect of Periostin on OPG expression.

Since, in our study, Periostin inhibition was achieved by means of FAK inhibition, it is likely that other FAK-dependent proteins relevant to osteoblast differentiation are also inhibited, so that the effect investigated is not solely, but only partially, attributable to Periostin. For example, fibronectin acts similarly to Periostin via FAK activation [94,95,96] and is also involved in osteoblast differentiation [97]. However, the most important regulator of osteoblast differentiation, Runx2, does not act via FAK [98] and is, therefore, not affected by the used antibodies.

## 4. Materials and Methods

### 4.1. Patient Samples and Group

Primary osteoblasts were isolated from the femoral heads of patients who had undergone femoral head replacement surgery and osteodensitometry using dual-energy X-ray absorptiometry. Both measures were medically indicated, diagnosis and therapy were at no time restricted by the study, and patients consented to further use of the femoral heads and measurement data. All femoral head removals were performed at the Department of Orthopaedics and Trauma Surgery, University Hospital Düsseldorf.

Osteoblasts from 29 subjects were used for the results of this study. Table 1 provides an overview of selected epidemiological data for each subject and indicates the bone mineral density measured. Of the 29 subjects, 19, or approximately two-thirds, are female and 10, or approximately one-third are male. Femoral heads were obtained between April 2018 and March 2023.

Figure S7a illustrates the age distribution as a box and whisker plot. The minimum, median, and maximum are marked. All patients were between 54 and 97 years old, with an age median of 75 years old.

Categorisation into normal and osteoporotic bone density groups was based solely on the T-values of the DXA scan, according to the WHO threshold classification, without regard to disease manifestation [5]. As DXA measurements of the spine and femoral neck were not available for all patients, the bone density of the femoral neck was always used first for categorisation. This was the lower value for all patients who had a measurement at both sites. Only when no femoral neck bone density data were available, such as after bilateral femoral head replacement, was the spine bone density used instead.

Figure S7b shows the numerical distribution of patients in the osteoporosis group and the group with normal bone density as a control according to gender.

### 4.2. Isolation of Human Osteoblasts

To isolate osteoblasts from surgically removed femoral heads, the cancellous bone was separated from the compacta using a sharp spoon. The fragments were transferred to a centrifuge tube into which 0.25% collagenase type IV (Life Technologies Ltd., Thermo Fisher Scientific, Waltham, MA, USA) in wash medium (1% Penicillin/streptomycin 10,000 U/mL/10 mg/mL in Gibco™ Ham’s F-12 Nutrient mix from Life Technologies Ltd., Thermo Fisher Scientific, Waltham, MA, USA) was sterile filtered. This mixture was incubated for 2.5 h at 37 °C with shaking; then, the collagenase solution was removed and transferred to a new centrifuge tube. This was centrifuged for 5 min at 400× *g*, and the supernatant was aspirated and resuspended with wash medium.

The pellet was then centrifuged again at 400× *g* for 5 min, and the supernatant was removed. Finally, the pellet was resuspended in standard cell culture medium, plated into two T75 cell culture flasks, and further treated as described below.

### 4.3. Cell Culture

Osteoblasts were incubated in cell culture medium (see Table S2) at 37 °C, 5% CO_2_, and 100% relative humidity, in the absence of light.

The cells were subjected to regular examination under a light microscope to ascertain their vitality, growth, and potential for contamination. The medium was replaced twice weekly, or more frequently in instances of light microscopic aberrations. Upon achieving a dense cell layer at the base of the cell culture flasks, the cells were passaged and distributed across multiple flasks.

### 4.4. Cell Culture Passage and Cell Count

The cells were transferred to a new container and/or divided into several containers simultaneously. Firstly, the cells were washed once with Dulbecco’s Phosphate Buffered Saline, modified, without Calcium, Chloride and Magnesium (PBS, Sigma-Aldrich Co., St. Louis, MO, USA). To detach the cells, 1× trypsin-PBS solution was added to cover all cells. They were then incubated for 5 min at 37 °C, 5% CO_2_, 100% relative humidity, and in the absence of light.

Subsequently, the cell detachment was checked under a light microscope. Not-yet-detached cells were detached manually using a cell spatula. To stop the reaction, cell culture medium was added. The suspension was then transferred to a centrifuge tube and subjected to centrifugation at 300× *g* for a period of five minutes. The supernatant was then aspirated, and the remaining cell pellet was resuspended in new cell culture medium. At this step, the cell number could be determined by counting in the Neubauer counting chamber and the suspension plated with the desired concentration achieved through dilution.

### 4.5. Cultivation and Differentiation During Experiments

All experiments were carried out in well plates. The incubation period for experiments with undifferentiated osteoblasts was consistently four days in a 6-well plate. On day 0, plating was conducted in a total of five duplicates at a concentration of 2.35 × 10^4^ cells/cm^2^, which corresponds to a concentration of 1.42 × 10^5^ cells/mL at 1.5 mL/well. On day 1, the cell culture medium was changed with the addition of the desired additive, and on day four, the supernatant was removed, cell activity was measured, and/or the cell pellet was isolated. The cytokines used are listed in Table S3.

To let the osteoblasts differentiate for 21 days, they were first incubated in cell culture medium in the corresponding plates until they had formed a cell lawn under the light microscope, indicating that contact inhibition had occurred, and they were adherent. Subsequently, the differentiation process commenced, with the differentiation medium (see Table S4) and additives (see Table S3) being replaced twice weekly.

The final change was made on day 17, ensuring that the medium was four days old at the conclusion of the experiment. The differentiation process was always accompanied by a control group of undifferentiated osteoblasts, which were used to measure cell activity and for alizarin red S staining. All differentiation experiments were conducted in 6-well plates, where supernatant was removed and immediately frozen for subsequent ELISA, cell activity was measured, and cell pellets were isolated for protein isolation, and in 48-well plates for alizarin red S staining. Experiments were conducted in duplicates in the 6-well plate and in quintuplicates in the 48-well plate. The mean value of the measured data was used in each case.

### 4.6. Cell Activity Measurement with CellTiter-Blue^®^ (CTB)

The CellTiter-Blue^®^ assay is employed to quantify cellular activity through the reduction of the dye resazurin by the cells. This alters the fluorescence maximum of the solution, which can be quantified photometrically. The fluorescence maximum is proportional to the cell activity, allowing for the estimation of cell numbers.

The osteoblasts were initially seeded on a well plate and incubated. After the excess cell culture medium was aspirated, 5% CellTiter-Blue^®^ Reagent (Promega Corporation, Madison, WI, USA) in cell culture medium was added to the wells. At this step, a well containing no cells was introduced as a control sample. Cells were incubated for one hour at 37 °C, 5% CO_2_, and 100% relative humidity, in a dark environment. Thereafter, 100 µL of medium from each well was transferred to the 96-well plate for photometric determination, with at least two wells containing the same solution to introduce controls from which an average value was calculated.

The photometric measurement was performed in the Victor X2 Multilabel Microplate Reader (filter: P540, F590, Perkin Elmer Inc., Waltham, MA, USA) at 590 nm.

### 4.7. Alizarin Red S Staining

To determine the degree of matrix mineralization by osteoblasts, alizarin red S staining was conducted in 48-well plates after a 21-day incubation with differentiation medium. The wells were initially washed with PBS. A total of 500 µL of ROTI^®^Histofix (Carl Roth GmbH + Co. KG, Karlsruhe, Germany) was added to each well, and they were incubated for 15 min at room temperature, which fixed the osteoblasts to the bottom of the plate. Then, all wells were washed with distilled water. Subsequently, the wells were incubated with 500 µL of 0.5% alizarin red S monosodium salt in distilled water for 20 min at 37 °C, in ambient conditions, in the absence of light.

The alizarin dye forms complexes with the calcium deposits formed by differentiated osteoblasts, which cannot be washed away with water. The wells were subjected to repeated washing with distilled water until the supernatant remained colourless. A final wash was conducted with PBS. A solution containing 10% cetylpyridinium chloride in distilled water was employed for redissolution, with 500 µL per well incubated for 2–3 h. Subsequently, the dyed supernatant was transferred to cuvettes, and the optical density was measured at 600 nm for a 1 cm light distance in the Bio Photometer with a 8,5mm light centre height (Eppendorf SE, Hamburg, Germany).

All measured values of alizarin red S staining were determined at least fivefold, and a mean value was calculated. In addition, the corresponding value of undifferentiated osteoblasts was subtracted from each value of differentiated osteoblasts to establish a baseline. The data were then normalised to the cell count determined in the CTB measurement of the corresponding well on the 6-well plate.

### 4.8. Nucleic Acid Analysis

Polymerase chain reactions of previously isolated RNA were used for the analysis of the Periostin splice variants expressed by human osteoblasts.

The RNA was isolated using the Trizol method on osteoblasts from 6-well plates. One mL of TRI Reagent^®^ was added to the cell pellet in the 1.5 mL reaction tube and incubated for five minutes at room temperature. Two hundred µL of chloroform was added, mixed, and incubated at room temperature for 10 min. Following centrifugation at 2500× *g* and 4 °C for 15 min, complete phases were formed. The upper transparent phase, which contains the RNA, was then transferred carefully to a new reaction tube. Next, 500 µL of isopropanol was added, thoroughly mixed, and again incubated for 10 min at room temperature, followed by centrifugation at 2500× *g* and 4 °C for 10 min. The resulting pellet was then washed twice with 1 mL ethanol (75%), and centrifugation was conducted at 2000× *g* and 4 °C for 7 min. Finally, the supernatant was removed, and the pellet was allowed to dry completely.

The resulting pellet, containing the isolated RNA, was diluted with RNase-free water. In order to optimise subsequent PCR results, purification was carried out using the DNA-free DNA extraction kit purchased from Invitrogen, Thermo Fisher Scientific, Waltham, MA, USA, according to the manufacturer’s instructions.

The concentration of the isolated RNA was determined utilising a NanoDrop 2000 spectrophotometer (Thermo Fisher Scientific, Waltham, MA, USA). The RNA was employed directly for reverse transcription (RT) or frozen at −80 °C for subsequent utilisation.

RT was performed using the Omniscript RT kit (QIAGEN GmbH, Hilden, Germany), with concentrations according to the manufacturer’s instructions. The reaction was carried out at 37 °C for 60 min in the T100 thermal cycler (Bio-Rad Laboratories Inc., Hercules, CA, USA). The resulting cDNA was stored at −20 °C for subsequent polymerase chain reaction (PCR) analysis.

The real-time polymerase chain reaction (qPCR) was achieved using the SYBR™ Green PCR Master Mix (Applied Biosystems, Thermo Fisher Scientific, Waltham, MA, USA). In accordance with the instructions provided by the manufacturer, a master mix was prepared for each primer pair at 0.4 µM primer concentration each.

qPCR was carried out in a 96-well plate, in accordance with a previously established layout, on the 7300 Real-Time PCR System (Thermo Fisher Scientific, Waltham, MA, USA) with the programme shown in Table S5. The measurements were taken following the completion of Phase 3, Step 2.

All samples were measured in triplicates. All measured values that were more than 0.5 cycles apart in the cycle threshold (CT) were excluded. An average value was calculated from the remaining 2 or 3 CT values. Finally, the 2^−ΔCt^ or 2^−ΔΔCt^ method was used for evaluation.

Furthermore, we evaluated GAPDH and β-actin as housekeeping genes in osteoblasts and normalised all qPCR results with β-actin as control.

For the detection of Periostin splice variants, primers according to Cai et al. were used as shown in Table 2 [47].

To verify our findings, conventional polymerase chain reaction (PCR) was performed using the Taq PCR Core Kit (QIAGEN GmbH, Hilden, Germany) and primers at 0.4 µM each. Subsequent gel electrophoresis was done with SYBR™ Gold nucleic acid gel stain (Invitrogen, Thermo Fisher Scientific, Waltham, MA, USA) at a ratio of 1:4 in a 2% agarose gel. PCR products were then eluted from the agarose gel using the QIAquick Gel Extraction Kit (QIAGEN GmbH, Hilden, Germany), in accordance with the manufacturer’s instructions. The samples were dispatched to Eurofins Genomics in Ebersberg, Germany, for sequencing. The results were subjected to analysis using the NCBI Nucleotide-BLAST database.

### 4.9. Western Blot Analysis

The expression of Periostin was analysed at the protein level in addition to the RNA level. Western blotting was employed for the detection of intracellular Periostin.

The cell pellets were isolated from the cell cultures in the 6-well plate in accordance with the procedures described above. Depending on the cell pellet size, the pellet was covered with 20–40 µL Radioimmunoprecipitation assay (RIPA) buffer (1 tablet in 10 mL cOmplete™ Mini protease inhibitor-Cocktail, Tris, pH 8.0, 150 mM sodium chloride, 0.5% Deoxycholic acid sodium salt (DOC), 1% Nonidet-P40 substitute (NP-40), 0.1% Sodium dodecyl sulphate (SDS)) and frozen at −80 °C.

The cell pellets were subjected to sonication using an ultrasonic processor (UP50H by Hielscher Ultrasonics GmbH, Teltow, Germany) with ten pulses at cycle 0.5 and an amplitude of 80%. This process facilitated the mechanical dissolution of the pellets.

The concentration in the lysate was determined with the Pierce™ BCA Protein Assay Kit (Thermo Fisher Scientific, Waltham, MA, USA), according to the manufacturer’s instructions. Samples were diluted in distilled water, with 20 µg of protein added to a 10 µL volume and an additional 4 µL of Laemmli buffer (Tris-HCl pH 6.8, 40% Glycerine, 8% SDS, 0.01% Bromophenol blue, 20% 2-mercaptoethanol in aqua destillata).

The samples were then subjected to centrifugation for one minute at 3000× *g* and room temperature, and subsequently denaturised on the heating block for five minutes at 94 °C. Gel electrophoresis was carried out as SDS-PAGE in 4–15% Mini-PROTEAN™ TGX Stain-Free™ protein gels, 15 wells, 15 µL (Bio-Rad Laboratories Inc., Hercules, CA, USA).

To ensure consistency in the subsequent immunolabelled blots, the total amount of protein in each lane was quantified using the ChemiDoc MP Imaging System (Bio-Rad Laboratories Inc., Hercules, CA, USA).

Proteins were then transferred to a nitrocellulose membrane in the Trans-Blot^®^ Turbo™ Transfer System (Bio-Rad Laboratories Inc., Hercules, CA, USA) at 2.5 A and 25 V for a period of 10 min.

The primary antibody was applied in concentrations specified in Table S6 in blocking solution (Tris pH 7.5, 150 mM sodium chloride, 0.1% Tween 20, 5% bovine serum albumin (BSA) in aqua destillata). The blot was then washed, immunolabeled with the secondary antibody, and once more washed in 1X Tris-buffered saline with 0.1% Tween^®^ 20 Detergent (TBS/T). Lastly, the blot was treated with Immobilon Forte Western HRP substrate (Merck KGaA, Darmstadt, Germany) and imaged using the ChemiDoc MP Imaging System. The image analysis was conducted in ImageLab 6.1.0 build 7 (Bio-Rad Laboratories Inc., Hercules, CA, USA), with the data normalised to the total protein content of the respective gel lane.

### 4.10. ELISA

In the ELISA, the secreted proteins of the osteoblasts into the extracellular space were detected in the cell culture medium. To this end, the cell culture medium was removed after three days of incubation for undifferentiated osteoblasts and four days of incubation for differentiated osteoblasts, and was placed immediately on ice in 1.5 mL SafeSeal tubes and frozen at −80 °C.

The OPG, IL-6, and Periostin ELISA was conducted in accordance with the instructions provided by the manufacturer (all R&D Systems, Minneapolis, MN, USA), utilising the requisite materials from the ancillary kit (R&D Systems, Minneapolis, MN, USA). The measurement was conducted using the Victor X2 Multilabel Microplate Reader at a wavelength of 450 nm. A further measurement at 540 nm was subtracted from this to facilitate manual correction of any potential plate inaccuracies. The data were normalised to the standard using GraphPad Prism (version 9.5.1 (733) from GraphPad Software, Boston, MA, USA), employing a logistic regression with four parameters.

### 4.11. Statistical Analysis

The statistical significances were analysed using GraphPad Prism. A significance level of 5% (*p* < 0.05) was selected to ensure a Type I error rate of α = 0.05. The level of significance is indicated by one star, two stars for *p* < 0.01, and three stars for *p* < 0.001. In instances where no statistically significant result was observed, the data were labelled as ‘ns’. All bar charts are presented as means with the corresponding standard deviations.

When comparing two groups, the appropriate statistical test was selected based on the normality of the data and the presence or absence of pairing. The tests employed were the *t*-test, the *t*-test with Welch’s correction, the Mann–Whitney U-test, and the Wilcoxon matched-ranks test.

The regression analyses for Periostin mRNA expression were tested for significance using a simple linear regression model.

## 5. Conclusions

Periostin-4 is dominantly expressed in osteoblasts. There is no change in splice variant expression in osteoblasts in osteoporosis conditions.

We found an altered relationship between Periostin expression and secretion when categorising patients with osteoporosis and normal bone density. We attributed this to a possible unknown post-translational modification affecting Periostin secretion in osteoblasts from osteoporotic patients. This modification could be caused by IL-6, although the exact mechanism and signalling of IL-6 in this context is unknown.

According to the current literature, Periostin is essential for bone regeneration [99], and a significant impact of Periostin in osteoblast differentiation could be demonstrated. While our findings support that Periostin is necessary for osteoblast differentiation, we found that it inhibits OPG secretion in osteoblasts, probably promoting bone resorption. The mechanism of this effect remains unknown. Interestingly, we were able to demonstrate this effect for all osteoblasts, not just those from osteoporotic donors. Periostin inhibiting OPG secretion has not been described before and raises new questions about the relationship between Periostin and OPG in healthy bone.

We attributed these contradictory results to the different functions of Periostin in the context of fracture healing. This secretion or possible post-translational modification could prevent the formation of a functional ECM by Periostin.

The bone-resorbing properties of Periostin and its interaction with IL-6 open up new possibilities for the pharmacological treatment of osteoporosis. Inhibition of both IL-6 and Periostin itself may prove to be viable options, although the exact effects on bone density are not yet clear and are likely to be dose-dependent.

It would be interesting to be able to assign the pathomechanisms of osteoporosis that we have shown to the high and low turnover variants of osteoporosis. This could be done by determining already established bone turnover markers in the blood of patients [100]. A significant correlation with Periostin in serum has been demonstrated for the bone formation marker PINP [52]. It is questionable whether this correlation exists in osteoporosis, when Periostin may no longer be involved in ECM formation and collagen cross-linking.

## Figures and Tables

**Table 1:** Bone mineral density (BMD) measurements of all patients.

#	Age (Years)	Sex (m/f)	BMD Femur (T-Value)	BMD Spine (T-Value)
1	85	F	−3.4	−0.3
2	79	F	−2.7	−2.1
3	77	F	−0.3	1.8
4	81	F	−3.7	−2.1
5	78	F	0.2	2.5
6	56	M	0.1	−0.7
7	69	F		1.5
8	55	F	−0.8	1.1
9	88	F	−3.9	−3.1
10	57	M	−2.6	−2.5
11	84	M	−4.1	−4.4
12	68	F	−1.8	−1.9
13	57	M		6.8
14	94	M	−2.6	−2.8
15	83	F		0.6
16	70	F		0.1
17	97	F		−3.4
18	54	F	0.6	
19	75	F	−2.7	−2.9
20	69	F	0	0
21	79	M	−1.2	1.6
22	86	M	−2.7	1.6
23	63	M		−2.8
24	60	F		2.5
25	66	F		0.2
26	77	F		−0.8
27	79	M	1.9	1.3
28	57	M	−3	−2.8
29	61	F	−2.6	−1.5

**Table 2:** Oligonucleotide primers used.

Gene	Primer Sequence 5′-3′	Manufacturer
GAPDH	Fwd: CCCTTCATTGACCTC Rev: ATGACAAGCTTCCCG	Eurofins Genomics, Ebersberg, Germany
Periostin (all isoforms)	Fwd: CAACGCAGCGCTATTCTGAC Rev: CCAAGTTGTCCCAAGCCTCA	Eurofins Genomics, Ebersberg, Germany
Periostin-1	Fwd: TGAAGGCAGTCTTCAGCCTA Rev: GTGACCTTGGTGACCTCTTC	Eurofins Genomics, Ebersberg, Germany
Periostin-2	Fwd: CCCGTGACTGTCTATAAGCC Rev: GTGACCTTGGTGACCTCTTC	Eurofins Genomics, Ebersberg, Germany
Periostin-3	Fwd: CCGTGACTGTCTATAGACCC Rev: TCCTCACGGGTGTGTCTTCT	Eurofins Genomics, Ebersberg, Germany
Periostin-4	Fwd: CCCGTGACTGTCTATAAGCC Rev: TCCTCACGGGTGTGTCTTCT	Eurofins Genomics, Ebersberg, Germany
Periostin-5	Fwd: CCGTGACTGTCTATAGACCC Rev: GTGACCTTGGTGACCTCTTC	Eurofins Genomics, Ebersberg, Germany
Periostin-6	Fwd: CCGTGACTGTCTATAGTCCTG Rev: ATTTGGTGACCTTGGTGACC	Eurofins Genomics, Ebersberg, Germany
Periostin-7	Fwd: CCGTGACTGTCTATAGTCCTG Rev: TCCTCACGGGTGTGTCTTCT	Eurofins Genomics, Ebersberg, Germany
Periostin-8	Fwd: TGAAGGCAGTCTTCAGCCTA Rev: TCCTCACGGGTGTGTCTTCT	Eurofins Genomics, Ebersberg, Germany
β-Actin	Fwd: GGATGCAGAAGGAGATCACG Rev: ATCTGCTGGAAGGTGGACAG	Biolegio B.V., Nijmegen, The Netherlands

## Data Availability

The raw data supporting the conclusions of this article will be made available by the authors upon request.

## Supplementary Materials

The supporting information can be downloaded at https://www.mdpi.com/article/10.3390/ijms26030932/s1.
