# Supplementary material for: Periostin Splice Variant Expression in Human Osteoblasts from Osteoporotic Patients and Its Effects on Interleukin-6 and Osteoprotegerin
Source: Int J Mol Sci. 2025 Jan 23;26(3):932. doi: 10.3390/ijms26030932 (PMC11816753; doi:10.3390/ijms26030932)
Supplement: Supplementary file 1 [file ijms-26-00932-s001.zip › ijms-3414087-supplementary.pdf]

International Journal of
Molecular Sciences

MDPI

Article

Periostin splice variant expression in human osteoblasts from osteoporotic patients and its effects on IL-6 and OPG

Till Kuebart [1], Lisa Oezel [1,*], Beyza Gürsoy [1], Uwe Maus [1], Joachim Windolf [1], Bernd Bittersohl [2], Vera Grotheer [2]

[1] Department of Orthopedics and Trauma Surgery, Medical Faculty and University Hospital Duesseldorf, Heinrich Heine University, Duesseldorf, Germany; lisa.oezel@med.uni-duesseldorf.de
[2] Department of Orthopedics, Medical Faculty of the University Hospital Ostwestfalen-Lippe, Bielefeld University, Bielefeld, Germany; vera.grotheer@uni-bielefeld.de
* Correspondence: lisa.oezel@med.uni-duesseldorf.de

Supplementary Materials:

1. *Tables*

Table S1. Periostin isoforms according to UniProtKnowledgeBase [36].

Isoform	UniProtKB#	Protein length (aa)	Skipped exons
1; full-length	Q15063-1	836	None
2	Q15063-2	779	17, 18
3	Q15063-3	781	17, 21
4	Q15063-4	751	17, 18, 21
5	Q15063-5	809	17
6	Q15063-6	749	17, 18, 19
7	Q15063-7	721	17, 18, 19, 21
8; Periostin-like factor	Q15063-8	808	21
9	Q15063-9	806	18
10	Q15063-10	778	18, 21

Table S2. Cell culture medium

Medium and additive	Final concentration
FBS Superior Supplemented RBS, Origin Brazil (FCS, Sigma-Aldrich Co, St. Louis, USA)	10 %
Penicillin/streptomycin 10000 U/mL/10 mg/mL	1 %
Ad Gibco™ Dulbecco's Modified Eagle Medium +4,5 g/L D-Glucose, L-Glutamine, -Pyruvate (DMEM, Life Technologies Ltd, ThermoFisher Scientific, Waltham, USA)	

Table S3. Concentrations of additives used

Additive	Catalogue number	Manufacturer	Concentration
Human IL-6	200-06	PeproTech, ThermoFisher Scientific, Waltham, USA	250 U/mL
Anti-IL-6	MA5-23698	Invitrogen, ThermoFisher Scientific, Waltham, USA	0,1 µg/mL
Human IL-8	200-08	PeproTech, ThermoFisher Scientific, Waltham, USA	100 U/mL

Reparixin L-lysine salt	6957	Tocris Bioscience, Bristol, UK	10^{-7} M
Human Periostin Protein	10299-H08H	Sino Biological Inc., Beijing, China	100 ng/mL
PF-573228	PZ0117	Sigma-Aldrich Co, St. Louis, USA	10 µM

Table S4. Differentiation medium

Medium and additive	Final concentration
FCS	10 %
Penicillin/streptomycin 10000 U/mL/10 mg/mL	1 %
Dexamethasone	500 nM
L-Ascorbic Acid 2-Phosphate Sesquimagnesium Salt	50 µM
Glycerin-2-phosphate	10 mM
Ad DMEM	

Table S5. Realtime polymerase chain reaction (qPCR) Cycler programme

Phase	Step	Repetitions	Temperature (°C)	Duration (s)
1	Start	1	50	120
2	Initial denaturation	1	95	600
3	Denaturation	45	95	15
	Annealing and Extension		60	60

Table 6. Concentrations of the primary and secondary antibodies used for western blotting

Antibody	Catalogue number	Manufacturer	Concentration
Mouse IgG, HRP-linked	7076	Cell Signaling Technology Inc., Danvers, USA	1:2000
Periostin [EPR19936]	ab219057	Abcam plc, Cambridge, UK	1:1000
Rabbit IgG, HRP-linked	7074	Cell Signaling Technology Inc., Danvers, USA	1:1000
Vitamin D Receptor (VDR)	sc-13133	Santa Cruz Biotechnology Inc., Dallas, USA	1:1000

2. Figures

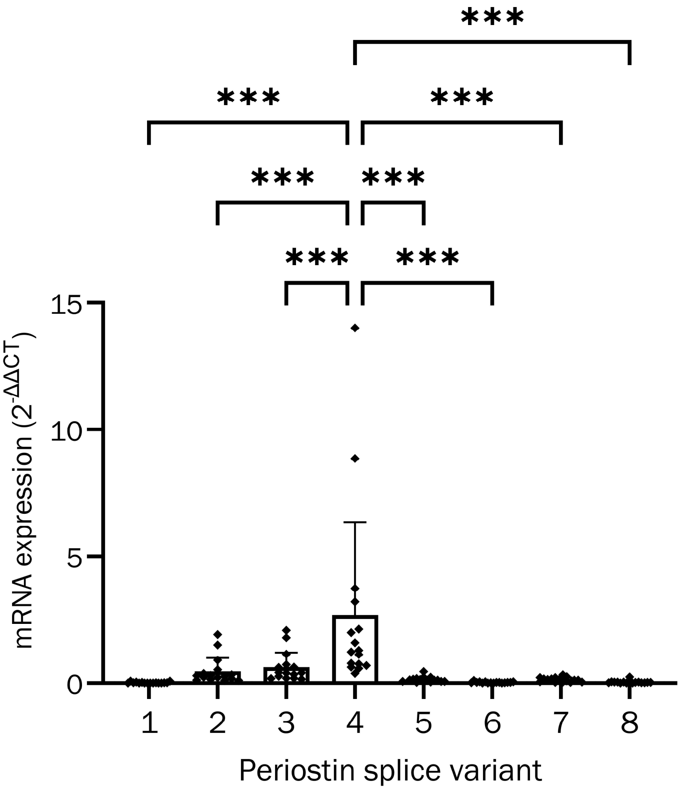

(**a**)

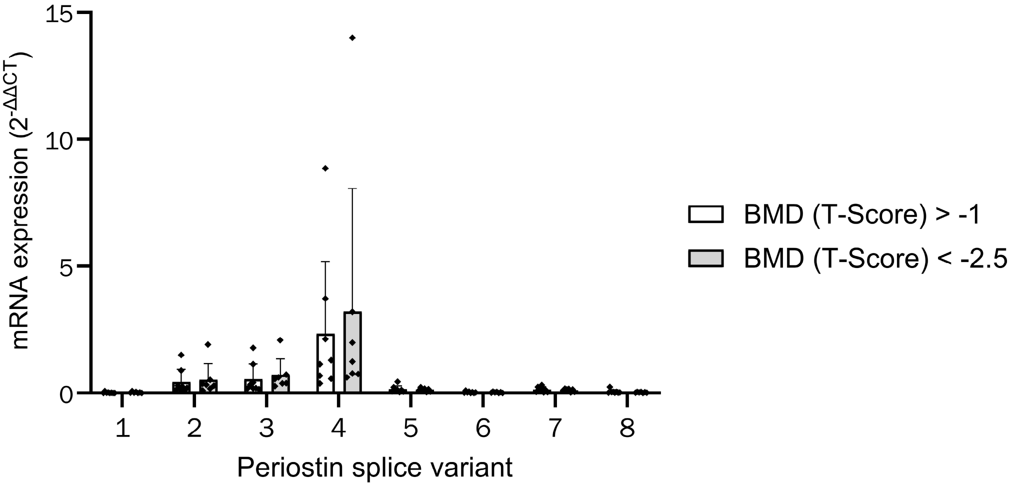

(**b**)

Figure S1. (**a**) mRNA expression of Periostin splice variants in osteoblasts relative to total Periostin expression plotting all data points (Single data points shown, Friedman test, Friedman statistic = 104.4, $p < 0.0001$, n = 16, with post-hoc analysis by Wilcoxon matched-pairs signed rank test with manual correction of the α-error according to Bonferroni, $p < 0.0007$, n = 16). (**b**) Comparison of mRNA expression of Periostin splice variants in osteoblasts relative to total Periostin expression between osteoporosis and normal bone density plotting all data points (multiple unpaired t-tests with Welch's correction, each $p > 0.3$, n = 15).

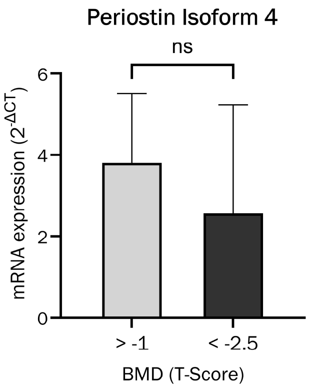

Figure S2. Comparison of relative Periostin isoform 4 mRNA expression between normal bone density and osteoporosis patients (Mann-Whitney test, U = 15, p = 0.2593, n = 14).

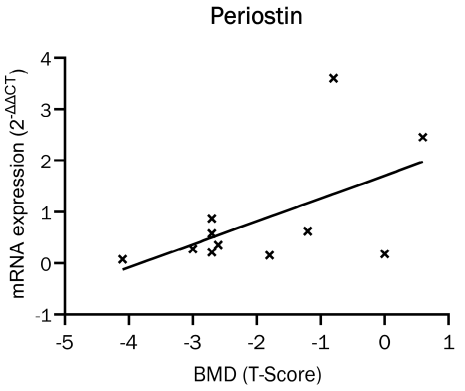

Figure S3. Relative Periostin mRNA expression in relation to bone density (simple linear regression, y = 0.4460x + 1.704, R^2 = 0.3116, p = 0.0743, n = 11).

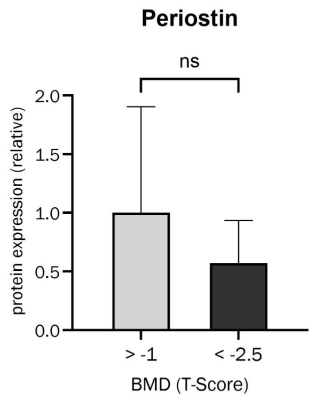

Figure S4. Comparison of relative Periostin protein expression between normal bone density and osteoporosis patients (Mann-Whitney test, U = 16, p = 0.5338, n = 13).

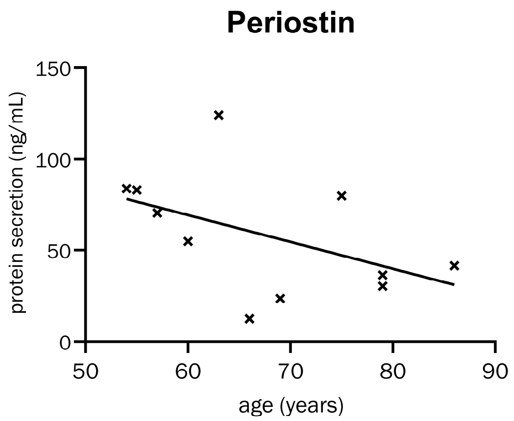

Figure S5. Periostin secretion in relation to patient age (simple linear regression, y = −1.475x + 157.8, R2 = 0.2349, p = 0.1309, n = 11).

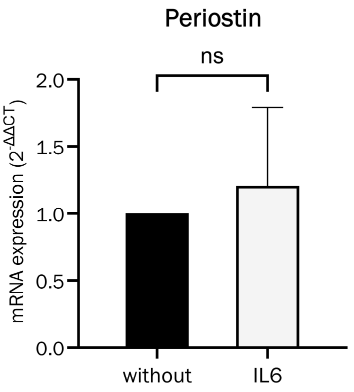

Figure S6. Effect of IL-6 on Periostin mRNA expression in osteoblasts (Wilcoxon matched-pairs signed rank test, p = 0.1726, n = 14).

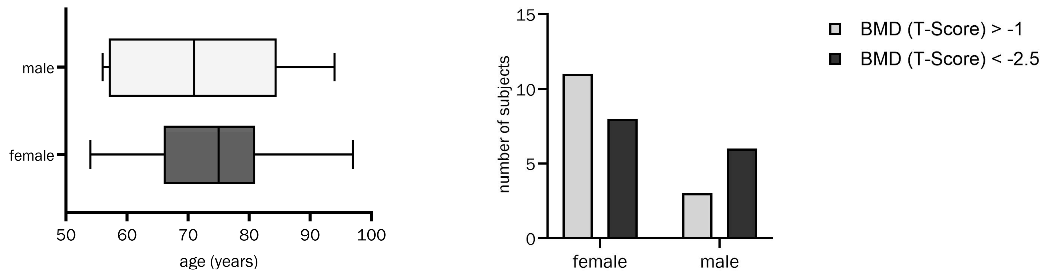

(**a**) (**b**)

Figure S7. (**a**) Age distribution by sex in box and whiskers plot. (**b**) Distribution of osteoporosis and normal bone density patients by sex.
